# English language ability as an exclusion criterion in emergency research taking place in the East Midlands, its impact on recruitment and a survey of the views of research professionals

**DOI:** 10.1186/s13063-026-09829-7

**Published:** 2026-06-15

**Authors:** Rachelle Sherman, Andrew Tabner, Graham Johnson

**Affiliations:** University Hospitals of Derby & Burton NHS Foundation Trust, Derby, UK

**Keywords:** English language, Communication, Underserved groups, Inclusivity in research, Clinical trial recruitment

## Abstract

**Background:**

English language ability is commonly used within eligibility criteria in research, despite limited strategies to assess it objectively. Improving inclusion of underserved groups in research is an important and ongoing priority for the research community, including funding bodies such as the National Institute for Health and Care Research (NIHR). We asked how excluding those who cannot communicate in English impacts recruitment in Trauma and Emergency Care trials taking place in the East Midlands (UK) and explored drivers behind including such eligibility criteria.

**Methods:**

We reviewed Trauma and Emergency Care studies with at least one site in the East Midlands, which completed in the 5 years prior to November 2020 and included English language ability within their eligibility criteria. We obtained screening data where available (numbers screened and excluded, and reasons for exclusion) to estimate exclusions due to language. We conducted an online survey of research professionals based in East Midlands’ Research & Development departments, Clinical Trials Units and Research Ethics Committees, to explore views on the use of this criterion.

**Results:**

We found that 18% of the 129 studies reviewed directly excluded those unable to communicate in English. Screening data were available for 5/15 studies included in the analysis; 2–7% of screened patients were excluded due to language. Those we surveyed showed an awareness of the ethical and scientific issues raised by this exclusion among respondents and perceived the drivers behind this exclusion to be linked to costs.

**Conclusions:**

A lack of available screening data meant that we were unable to determine the true scale of exclusion based on English language ability in publicly funded emergency medicine research in the East Midlands, but our findings indicate that it may impact on overall recruitment to studies, as exclusion rates were higher than that reported in the population. Given the importance of improving inclusivity and diversity in research, more work is needed to understand this at a national level and determine if strategies to overcome language barriers can support inclusivity and overall recruitment rates.

**Supplementary Information:**

The online version contains supplementary material available at 10.1186/s13063-026-09829-7.

## Background

It is widely acknowledged that many clinical trials struggle to meet their recruitment targets, with multiple factors impacting recruitment success [1, 2]. Language proficiency is regularly used within the eligibility criteria of research studies [3, 4] and is well recognised as a barrier to trial participation [5–7], but we found no quantitative data demonstrating how language-based exclusion criteria impact recruitment to studies. It is not clear whether the rate of exclusion is proportionate to the number of non-English speakers in the general population (approximately 1.8% of the population of England and Wales [8] and 1.9% in the East Midlands [9]) or whether recruitment is disproportionately affected. There are no strategies to support researchers in the objective assessment of language proficiency during the consent process [3, 10], potentially resulting in recruiters selecting only those patients who are fluent in English [5]. Studies in the UK are not always representative of their target population and ethnic minorities are frequently underrepresented [3, 11, 12]. Initiatives such as the National Institute for Health and Care (NIHR) INCLUDE project have highlighted the need to improve the inclusion of underserved groups, including those with language barriers [11, 13, 14]. For the purposes of this study, English language proficiency was considered broadly, including the ability to read, speak, hear and understand English, thereby encompassing those with speech, hearing or visual difficulties; however, these barriers are not explicitly distinguished within trial eligibility criteria.

We chose to focus on research projects taking place in Trauma and Emergency Care as this setting offers a unique set of challenges to research teams. Emergency departments are busy and time-pressured environments, making consent processes more challenging, particularly where interpreters are required. Whilst it is reasonable to expect that language barriers may feature in exclusion criteria, a preliminary unpublished literature search suggested a paucity of data exploring how this affects recruitment specifically in emergency medicine research [15].

The primary aim of this research was to evaluate the scale and potential impact on recruitment of excluding patients based upon their ability to communicate in English [5] using a sample of Trauma and Emergency Care research taking place in the East Midlands. Our secondary objectives were to better understand the rationale behind such exclusion by gathering views from those involved in the approval and delivery of clinical research in the East Midlands and then use the outputs from this study to inform a future, national project.

## Method

### Scale of the problem—review of research projects from the NIHR Trauma and Emergency Care (TEC) portfolio

Using the NIHR Open Data Platform (ODP), an NIHR portfolio management system, we identified research projects with at least one site within the East Midlands Clinical Research Network (CRN) catchment area (comprising Nottinghamshire, Derbyshire, Lincolnshire, Leicestershire, Rutland and Northamptonshire), irrespective of participation by additional sites outside the region, which had completed in the 5 years prior to November 2020. The geographic focus and 5-year completion window were selected pragmatically, reflecting local regional source of funding for this study, the need to maintain regional relevance, and available resources for data collection in late 2020. The projects included in the search met the definition of research, including randomised and non-randomised studies, and had appropriate ethical approval to be eligible for inclusion on the NIHR CRN portfolio [16]. We identified research studies that used eligibility criteria that included the terms “language”, “communic*”, or “English”. We used journal articles, study reports and contacted corresponding authors to find the numbers of potential participants in these studies excluded based upon language ability. We estimated a percentage of screened patients excluded, based on the information reported in each published paper, or provided by the authors. In one study, the authors could not provide precise numbers but instead provided an estimated percentage. Study anonymity is maintained in this manuscript, as its purpose is to evaluate a systemic exclusion rather than the methodological approaches of individual trials.

### Cause of the problem—focus groups

We contacted five Research Ethics Committees (RECs) within the East Midlands and asked them to circulate details of the study to their members, with an invitation to take part in a focus group to discuss the use of eligibility criteria to exclude those unable to communicate in English. We planned to conduct two focus groups with at least four participants per group; however, recruitment for this component proved infeasible, with only one positive response to participation despite offering voucher incentives and sending a follow-up email reminder. Given these recruitment constraints, this component of the study could not be progressed as planned and was not pursued further.

### Cause of the problem—cross-sectional survey of NHS research & development, clinical trials unit staff and research ethics committee members

We developed a 12-item survey using Microsoft Forms (Additional File 1) that included questions to determine the following: the respondent’s experience of the use of eligibility criteria based on English proficiency; their understanding, or perception, of the rationale behind its use; and their thoughts on the impact of use of such criteria.

The survey was tested within the study team and a small number of independent peer reviewers, who also provided feedback on the study protocol, with revisions made based on feedback to question composition and structure. Responses were only mandated for the 10 closed-ended questions, with an added 2 open-ended questions inviting respondents to provide further comments.

The survey was distributed across all 8 NHS Research & Development (R&D) departments of acute hospital trusts listed as Clinical Research Network East Midlands partners [17], the 3 Clinical Trials Units (CTUs) and 5 RECs within the East Midlands. A central contact at each organisation was asked to cascade information to their teams involved in the approval and/or delivery of research. The East Midlands was chosen as the region to align it with the geographical focus of the study. Participants completed the survey anonymously, assessed their own eligibility to participate and provided information on their previous involvement in Trauma and Emergency Care Research, experience in their role and the type of organisation they were based in. Inclusion in a prize draw was offered to encourage participation. As dissemination occurred internally within organisations and no record was available of the number or identity of individuals invited, it was not possible to determine the total number of eligible participants or calculate a response rate. The survey remained open for 6 weeks from the date of the first distribution email; no minimum sample size was predetermined. Responses were extracted to Microsoft Excel following survey closure and presented using descriptive statistics.

### Analysis of free-text responses to open-ended questions

The project as conceived involved a reflexive thematic analysis of survey free-text responses. However, due to the relatively limited responses, the data were conceptually thin. We therefore undertook a qualitative content analysis of open-ended survey results [18], systematically describing the meaning of our data set using categories in a coding process. Two authors (RS and AT) independently examined the responses, reviewing and re-reviewing the survey data. They then generated initial codes, finding meaningful extracts of text and grouping them into recurring categories. These categories were refined through discussion, associations between categories identified, and over-arching parent categories described and defined. These categories summarise perceived ethical, scientific, and practical issues associated with language-based exclusion criteria. The categories were reviewed against the data set to ensure they adequately described the content, were not contradicted by the data set as a whole, and did not leave significant meaning identified yet undescribed.

## Results

### Review of research studies from the Trauma and Emergency Care (TEC) portfolio

**Fig. 1:**
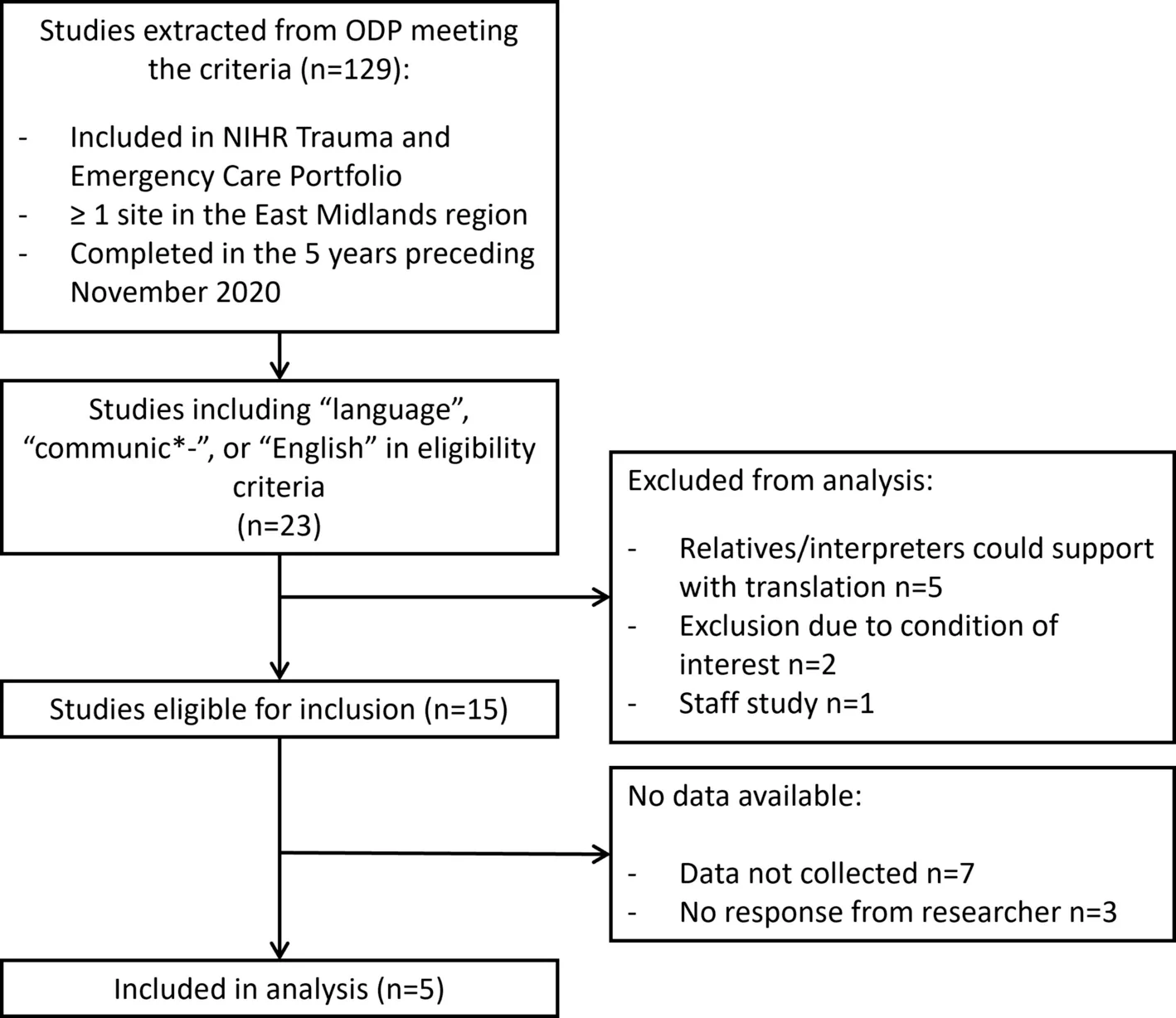
Flowchart showing inclusion and exclusion of studies from analysis

We identified 129 TEC studies that completed in the 5 years prior to November 2020, with 23 (18%) containing eligibility criteria that may have excluded those unable to communicate in English (Fig. 1). This is based on our search of criteria using terms “communic*”, “language”, or “English”. Examples of such criteria were “non-English speakers” and “participants must be able to communicate in English”. Studies that stated in their eligibility criteria that relatives or interpreters could support with communication difficulties (*n* = 5), staff studies (*n* = 1), or where communication was related to the condition of interest, e.g. traumatic brain injury (*n* = 2), were not included in the analysis of recruitment.

Only 1 of the 15 studies included in the analysis of recruitment, presented screening data in the primary publication. We contacted the remaining 14 corresponding authors, 11 of whom responded. Screening data were available for 5 of the 15 studies; data were not available for the other studies: not collected (*n* = 7); no response from corresponding author (*n* = 3).

Where data were available, 2–7% of screened patients were excluded due to language or communication. Three of the five studies did not meet their recruitment target, or progress beyond their internal pilot (Table 1).

**Table 1:** Studies that provided data on exclusion of non-English speakers

Study	Total screened	Excluded (*n*, %)	Actual/estimated	Comments
1	1903	41 (2%)	Actual	Over recruited, completed on final planned end date
2	211	12 (6%)	Actual	Completed on final planned end date
3	-	Unk, (5%)	Estimated	Did not meet target
4	470	35 (7%)	Actual	Did not progress beyond internal pilot
5	546	16 (3%)	Actual	Did not meet target

### Survey of research professionals

There were 31 respondents to the survey: R&D *n* = 16; CTU *n* = 14; REC *n* = 1; 21 (67%) had more than 5 years’ experience in clinical research, and 12 (39%) had worked on TEC studies.

Most respondents (20, 64.5%) said that they had worked on a study that included eligibility criteria based on participants’ understanding of English, and half (10, 50%) of these included TEC research studies. Most respondents (16, 67%) who had worked on studies with eligibility criteria based on participants’ understanding of English described the criterion as English not being the participants’ first language, rather than due to literacy or other communication issues.

**Fig. 2:**
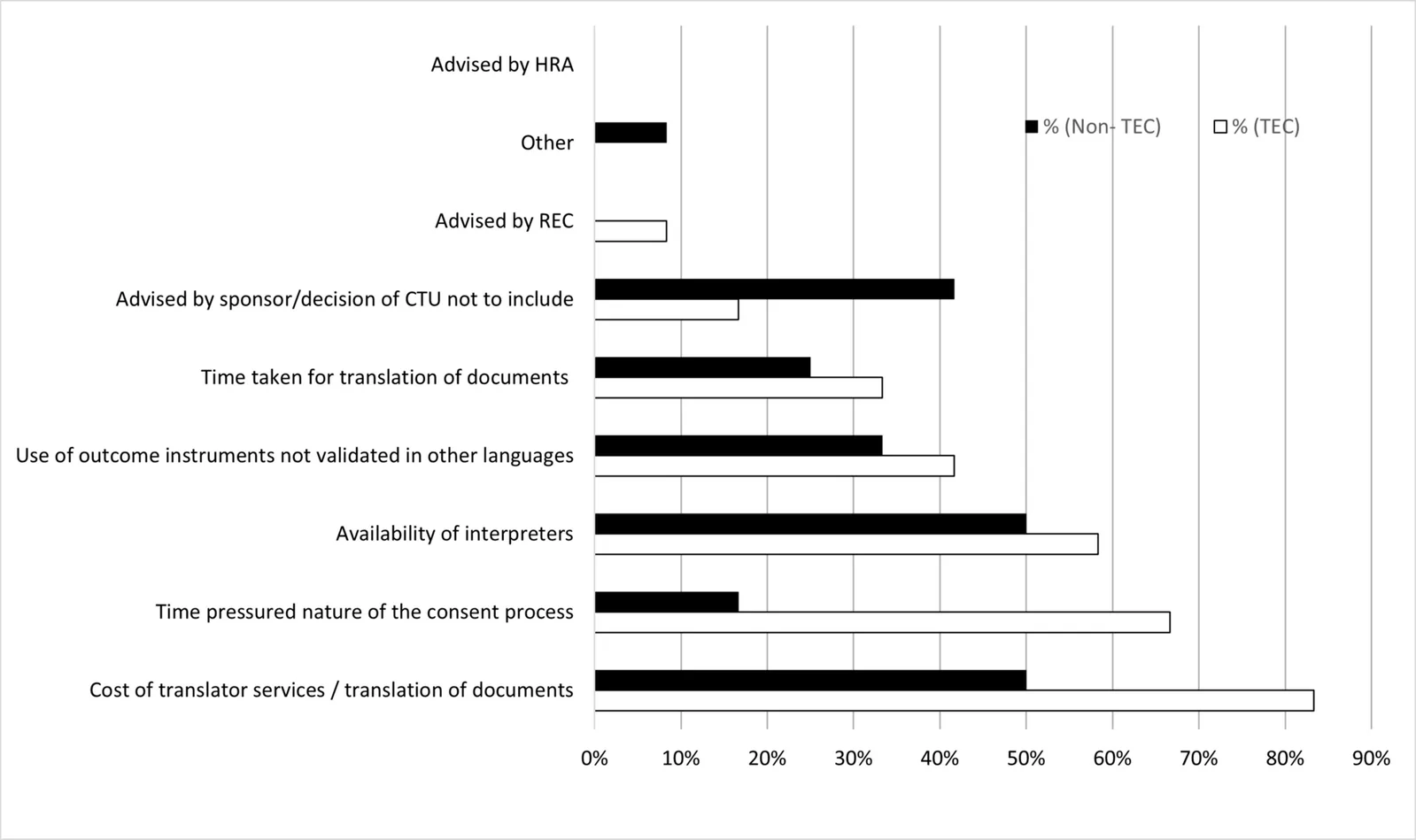
Bar chart showing the distribution of selected reasons for exclusion, categorised according to declared involvement in Trauma and Emergency (TEC) studies

Respondents were asked to choose reasons why they believed the study may have included this exclusion criterion (Fig. 2). There was a broad distribution of answers, but cost was the most often selected across both TEC (10, 83%) and non-TEC groups (6, 50%). This was followed by the time-pressured nature of consent process for those involved in TEC studies (8, 67%) and availability of interpreters for those not involved in TEC studies (6, 50%).

For the question “Do you believe that exclusion of these participants raises any issues?”, 22 (71%) answered “yes” and 8 (26%) answered “maybe”, but in response to question 11, few were “aware of initiatives to improve access to research for those who cannot understand English” (7, 23%).

### Analysis of free-text responses to open-ended questions

Analysis of the free text responses to the open-ended questions identified several recurring categories relating to perceived ethical, scientific, and systemic implications of excluding individuals unable to communicate in English. Given the heterogeneity of respondents’ roles, and their varied involvement across the research pathway, findings represent informed professional perspectives rather than definitive accounts of decision-making within individual studies.

### Categorisation of free-text responses

#### Category—exclusion of specific cohorts from research

This category captures responses indicating that the use of language-based exclusion criteria may result in the systematic exclusion of certain groups, including those with protected characteristics. Respondents raised ethical concerns of fairness and inclusivity, and were concerned regarding implications on generalisability and reliability of results.

#### Sub-category: different cohorts excluded

Description: Many respondents highlighted the underrepresentation of ethnic minority groups in research and acknowledged that other groups with communication difficulties may also be affected.

Illustrative quotes: “It’s probable that a proportion of non-native English speakers who are eligible for clinical trials are from ethnic minorities, which are underrepresented in clinical research”, “many members of the BAME community are excluded from research”, “excludes a relevant cohort of the population”, and “…some disabled people would have been less likely to be included (e.g. people with dyslexia and possibly stroke victims, or people with other movement disorders)”.

#### Sub-category: ethical implications

Description: Respondents highlighted the ethical implications of excluding specific cohorts from research.

Illustrative quotes: “Everyone should be given the opportunity to take part in research”, “It is unfair to exclude non-English speakers from research”, “not everyone is given the same opportunity to engage in research” and “all individuals should have access to research studies and be able to take part”.

#### Sub-category: impact on generalisability

Description: As well as ethical implications, many respondents raised concerns around the reliability and generalisability of results, acknowledging the scientific implications of the exclusion.

Illustrative quotes: “risk that the sample in the trial is not representative to the population of interest. Results might not be able to be generalised”, “reduced diversity in trials could lead to a lack of efficacy of research treatments in untested populations”, “If only English-speaking participants are included, can results really be generalised to the wider population” and “Language exclusions are very restrictive in truly attaining a representative sample of the target population”.

#### Category: systemic problem

Captures responses that suggest operational issues and inconsistent approaches across different trials. There was a suggestion of a lack of engagement from central research teams to support inclusion, often left to teams to manage this locally.

#### Sub-category: document translation

Description: Respondents commented on access to translated documents (and interpreters), but whilst some respondents claimed they were able to obtain translated documents easily, others were not.

Illustrative quotes: “translations are always available if necessary”, “Patient facing documents were only available in English” and “I have worked on a research study where interpreters were used successfully to recruit non-English speaking patients. Also, having patient information sheets available in other languages is very useful”.

#### Sub-category: funding availability

Description: Fewer respondents directly acknowledged funding as a reason for lack of strategies to include those who cannot communicate in English. Onus was put on sites to facilitate interpreters with no financial support.

Illustrative quotes: “There was no funding for interpreters, and we were not allowed to use relatives to translate”, “Funders could develop guidance similar to what INVOLVE is providing but covering costs and ideas for including these populations” and “all I found were suggestions about using hospital staff to translate”. 

#### Sub-category: disengagement of researchers

Description: Two respondents expressed that researchers may be unwilling to support inclusion of non-English speakers.

Illustrative quotes: “It is often, in my opinion, used as an excuse however not to do the work required to expand the inclusion criteria appropriately”, "Inability or unwillingness to provide information in languages other than English…it is not even just small-scale studies that do this—even multinational companies sometimes refuse, saying that questionnaires are “not validated” in other languages”.

## Discussion

We found that 23 of the 129 (18%) Trauma and Emergency Care (TEC) research studies we sampled used eligibility criteria that would exclude those unable to communicate in English without support from an interpreter or due to the condition of interest. Among these studies, screening data were limited, with only five studies explicitly recording these exclusions, reducing the ability to determine the true extent of this exclusion. Of these studies, 2–7% of potential participants were excluded due to limited English proficiency; this is higher than the 1.9% of the population that do not speak English as a first language in the East Midlands (UK census data) [8, 9] indicating a potential systematic exclusion that may impact on trial result generalisability, perpetuation of health inequalities, and trial recruitment rates. Not all studies for which screening data were available failed to meet their recruitment targets, and the extent to which language-based exclusion contributed to recruitment challenges cannot be determined from the available data. There are also multiple contextual factors to consider, including differences in the population, and other study eligibility criteria that may contribute to recruitment challenges.

Research professionals expressed an awareness of the ethical and scientific importance of including individuals with limited English proficiency in research, alongside recognition of the substantial practical challenges associated with facilitating their participation. Their perspectives spanned roles across sponsorship, governance, and trial delivery, meaning that views captured reflect a range of involvement in eligibility decisions rather than a single vantage point.

The CONSORT 2025 guidelines and their accompanying Explanation and Elaboration recommend that, where available, the number of individuals assessed for eligibility and reasons for exclusion prior to randomisation should be reported [19]. However, we found that in practice, exclusions occurring at the screening or pre-consent stage are not consistently collected or reported in trial publications. This represents a particular delivery challenge to be addressed in TEC research: screening in the emergency setting may require review of e.g. an entire emergency department patient population in real time, and capturing screening of this many patients is infeasible; where potential participant identification finishes, and screening/eligibility assessment starts, is sometimes difficult to articulate in practice.

We found that the proportion of potential participants excluded due to language ability (2–7%) exceeded the proportion of the general population reporting that they could not speak English (or Welsh) well or at all, which was 1.9% in the East Midlands according to the 2021 UK Census [8, 9]. This may be because there is no standardised approach to assessing proficiency, resulting in unnecessary exclusion based on subjective recruiter judgement [3, 5, 10]; equally, those who do not speak English well may be comparatively over-represented in the cohort seeking emergency care. [20] Whilst we found that criteria directly excluding participants based upon communicating in English were only explicit in 18% of studies sampled, we suspect that the common use of more general criteria such as "ability to provide informed consent" may result in a higher proportion indirectly excluded due to language barriers and explain why more than half of the research professionals we surveyed had encountered studies that excluded based on language ability. This is supported by previous work by Hussain-Gambles et al. [5], which found that recruiters in the NHS not only struggled to source interpreters, but they were also concerned about interpreters’ abilities to communicate clinical trial concepts, and therefore their ability to obtain truly informed consent.

As well as availability of interpreters, we found a commonly cited reason for the exclusion of those unable to communicate in English was funding constraint. This may be a false economy, as widening access to research and therefore the pool of potential participants could help studies reach their recruitment targets sooner, and subsequently reduce costs associated with study extensions. A recent NIHR-funded trial delivered by this study’s research team [21] excluded those unable to communicate in English due to a lack of funding available for document translation or use of interpreters, and the fact that some outcome measures were only validated in English. The team established that without this exclusion criterion, if approached patients had otherwise been eligible to take part and had agreed to do so, they could have represented 16% of the consented participants and the trial may have met its recruitment target sooner. The apparent cost savings of not providing language services contributed to the requirement for a funded extension, and a significant proportion of patients with the condition under investigation were denied the opportunity to participate in research, potentially affecting the generalisability of the results.

Previous work on the views of health professionals also identified a lack of translated documents as a barrier to the recruitment of non-English-speaking ethnic minorities [5], which we also found in our study. The financial implications of providing translated documents and interpreters were widely recognised, along with a lack of consistency from central research teams. Some respondents reported that they found it easy to obtain translated documents, while others were expected to use their local resources with no additional funding provided. Together, these findings highlight the tension between recruitment success and resource constraints, suggesting that short-term cost considerations may ultimately hinder recruitment by reducing the available participant pool. Such variability reflects a period in which responsibility for inclusion was not always centrally mandated or adequately funded by sponsors. It is important to contextualise these findings as much of this work pre-dated the introduction of the NIHR INCLUDE initiative and related policies, which now place explicit requirements on researchers to justify, resource and report inclusive recruitment strategies. Certainly, the costs for translating information sheets could be met centrally to serve the entire study, which could make a real difference to sites. Costs and interpreter availability may vary across geographical locations and clinical settings, as well as according to the breadth of languages spoken. We contacted our local NHS Trust Facilities and Management Team to retrieve details on the costs of services including document translation and provisions of interpreters (Additional File 2). Reviewing these alongside example costs provided by Trial Forge guidance [22] highlights that costs can vary greatly depending on the organisation, languages required, length of documents and formatting or proofreading requirements. Research costs are increasing year on year [23], and fierce competition calls upon researchers to be increasingly cost-effective. [5] The introduction of recent funder inclusion policies provides the opportunity to ensure studies are adequately resourced to support the inclusion of individuals with limited English proficiency.

One of the most common reasons for use of language-based exclusion in TEC research was the time-sensitive nature of the consent process; the use of deferred consent may therefore present a solution. Although the use of deferred consent, or “research without prior consent” (RWPC) has varying levels of acceptability amongst researchers [24], it is recognised that emergency research must not delay intervention; where requiring interpreters would introduce such a delay, use of a deferred consent model may facilitate inclusion. Interpreters could then facilitate a consent discussion once the time-critical stage had passed. The views of those from underserved groups, and particularly ethnic minority groups, must be sought on this; previous research highlights a lack of adequate representation from these groups, who may hold more conservative views on this approach [24, 25]. Care must be taken to avoid worsening a recognised mistrust of healthcare research [5, 6, 12] and co-development of such consent models in research is crucial.

Although language spoken is not itself a “protected characteristic”, it forms part of one’s nationality and ethnic or national origins, and therefore “race” [26]. Studies have highlighted that language barriers may disproportionately affect ethnic groups [5, 27], raising concerns regarding indirect discrimination where exclusion is not proportionate to the research aim [26]. While participant-level data on race and ethnicity were not available for the studies included in this review, these considerations draw on established evidence linking language proficiency with race and ethnicity, rather than direct measurement within the included studies. It is also important to recognise that exclusions attributed to English language ability may, in practice, encompass other communication barriers, including speech, hearing, or visual impairments. Due to variability in how eligibility criteria are interpreted and applied by recruiters, and limitations in the detail recorded within screening logs, it was not possible in this study to reliably distinguish exclusions arising specifically from limited English proficiency from those related to other communication impairments. Where a patient is unable to communicate in English based on a disability, for example individuals who are d/Deaf, failure to provide appropriate resources, such as sign language interpreters, risks unfair exclusion and indirect discrimination. Young et al (2016) discuss the complexities of recruiting the Deaf community but emphasise the ethical imperative to ensure that d/Deaf people who sign are provided with optimum resources to support informed consent [28]. Likewise, Good Clinical Practice (GCP) guidelines also direct that language used for study information should be understandable to the subject or the subject’s legally acceptable representative [29]. Taken together, these considerations align with the core ethical principles of the Declaration of Helsinki, particularly the need to ensure fair distribution of the burdens and benefits of research and respect for individual autonomy through meaningful informed consent.

We acknowledge that, beyond consent, study participation often requires completion of patient-reported outcome measures that are only validated in English, or in a small number of languages that may not be compatible for participants. A simple translation of such outcome measures (and indeed the information sheets and consent forms themselves) is frequently not appropriate, and some form of cross-cultural adaptation is required [30–32].

This study has provided us with valuable information to take forward into a future project. Whilst obtaining screening data to determine the scale of the exclusion was more difficult than we envisaged, the insight gained from the survey of research professionals has proved invaluable to determine next steps. A larger, national survey, with more comprehensive qualitative methods may provide a more complete picture of the attitudes within the research community and provide strategies to overcome the barriers that result in the use of exclusion based on ability to communicate in English.

### Limitations

The time lag between data collection in late 2020 and publication may limit the applicability of the findings to current research delivery contexts, particularly considering recent inclusion policies brought in by the NIHR. A lack of available screening data and inability to conduct focus groups meant that realising this study’s primary aim was challenging. It is interesting to note that even among those involved in research infrastructure delivery (REC members), engagement in research as participants was poor, with insufficient respondents to facilitate a focus group. As the survey was disseminated via organisational gatekeepers, a response rate could not be calculated limiting assessment of coverage and representation. This may have also contributed to the small number of responses we received. The small sample size, and geographic location, for the survey is a limitation of this study, and we acknowledge that other qualitative methods might have been more appropriate; however, these were outside of the scope of this study.

## Conclusion

This study demonstrates that use of language-based exclusion criteria in research, whilst not particularly common, may be disproportionately excluding cohorts of patients, and research professionals are concerned about the ethical and scientific impacts. A lack of data meant that we were unable to determine the true scale of exclusion based on English language ability in publicly funded emergency medicine research in the East Midlands, but our findings indicate that it may impact on overall recruitment to studies. Several reported reasons for use of these criteria exist, and not all can be addressed by additional funding. Research funders are actively encouraging researchers to improve the diversity of their study populations; there remains an important need to address the financial barriers present within the research funding system to support researchers in this goal.

Given the importance of improving inclusivity and diversity in research, more work is needed to understand this at a national level and determine if strategies to overcome language barriers can support inclusivity and overall recruitment rates.

## Supplementary Information

Supplementary Material 1. Is a copy of the Microsoft Forms survey.

Supplementary Material 2. Is the information on costs for provision of interpreters and document translation services provided by University Hospitals of Derby and Burton NHS Foundation Trust.

Supplementary Material 3.

## Data Availability

The datasets used and/or analysed during the current study are available from the corresponding author on reasonable request.

## Abbreviations

NIHR: National Institute for Health and Care Research
ODP: Open Data Platform
REC: Research Ethics Committee
R&D: Research & Development
CTU: Clinical Trials Unit
TEC: Trauma and Emergency Care
NHS: National Health Service
BSL: British Sign Language
RWPC: Research Without Prior Consent
BAME: Black, Asian and minority ethnic
GCP: Good Clinical Practice
PIS: Patient information sheet
HRA: Health Research Authority
CRN: Clinical Research Network
