# Supplementary material for: English language ability as an exclusion criterion in emergency research taking place in the East Midlands, its impact on recruitment and a survey of the views of research professionals
Source: Trials. 2026 Jun 15;27:543. doi: 10.1186/s13063-026-09829-7 (PMC13495170; doi:10.1186/s13063-026-09829-7)
Supplement: Supplementary file 1 [file 13063_2026_9829_MOESM1_ESM.pdf]

Additional File 1

NHS
University Hospitals of
Derby and Burton
NHS Foundation Trust

Survey: English language requirements as a barrier for participation in research.

A survey of R&D and CTU staff across the East Midlands to determine the extent to which an ability to communicate in English language proficiency impacts on recruitment into research studies in the East Midlands, by jointly evaluating qualitative and quantitative data.

IRAS: 292282
v1.1 16/Feb/2021

* Required

Participant information

We are conducting a research project to gather information on how requirements for a minimum level of understanding of the English language affect recruitment to research studies in the East Midlands. The following survey is intended for completion by staff members at Research & Development (R&D) departments at NHS Trust and at Clinical Trials Units (CTUs) in the East Midlands who are involved in the setup and delivery of research studies.

The survey should take no longer than 10 minutes to complete.

We intend to use the results of the survey to understand why and how often this exclusion criterion is included for research studies. We would also like your opinion on whether you believe this is a barrier to recruitment into research or otherwise impacts on the research you have been involved in. We will use the information to develop an application for a larger study that reviews this in more depth.

Everyone who completes the survey will be able to enter a draw for a chance to win a £20 Amazon voucher, if they wish to do so. The vouchers cannot be exchanged for cash and will be emailed to the winners. To enter the prize draw, you will need to provide an email address to us. We will only use this to contact you if you are a winner, so we can send you the voucher.

You do not have to take part in this research and you can change your mind at any time during or after the survey, without giving reason, but we will keep the answers to the survey for the analysis. If you have provided an email address, we will remove this from our records and you will not be entered into the prize draw

We will not collect any other identifiable information and will only keep your email address for as long as we need to undertake the draw. We will keep all information about you safe and secure. Once we have finished the study, we will keep some of the data (not your email address) so we can check the results. We will write our reports in a way that no-one can work out that you took part in the study. For further information on how we use your information, please see: https://www.uhdb.nhs.uk/research-how-we-use-your-information (https://www.uhdb.nhs.uk/research-how-we-use-your-information).

University Hospitals of Derby & Burton NHS Foundation Trust are the Sponsor for this study, and it has been funded by the East Midlands Clinical Research Network (CRN).

If you have any questions about the survey or how the data collected will be used, please contact rachelle.sherman@nhs.net (mailto:rachelle.sherman@nhs.net).

1. Have you read the participant information in the previous section? *

◯ Yes

◯ No

2. Are you happy to take part in the online survey? *

◯ Yes

◯ No

3. Do you work in a NHS R&D Department, Clinical Trials Unit or NHS Research Ethics Commitee based in the East Midlands?
This includes Nottinghamshire, Derbyshire, Lincolnshire, Leicestershire, Rutland and Northamptonshire *

◯ NHS R&D Department

◯ NHS Research Ethics Committee

◯ Clinical Trials Unit

4. Are you involved in studies in Trauma and Emergency Care Research? *

◯ Yes

◯ No

5. How long have you worked in clinical research? i.e. have been involved in the design, approval, management or delivery of research studies *

◯ 0-2 years

◯ 3-5 years

◯ 5-10 years

◯ More than 10 years

6. Have you ever worked on a study that includes eligibility criteria based on a participant's understanding of English? ("Worked on" may include designed, approved or supported in the management and/or delivery.) *

◯ Yes, this includes Trauma and Emergency Care research I have worked on

◯ Yes, this does not include Trauma and Emergency Care research

◯ No

◯ Not sure

7. To your understanding was this based on English not being their first language, or for other reasons they may be unable to communicate in English (e.g. difficulty hearing or reading)? *

- ◯ English not being their first language
- ◯ Being unable to communicate for other reasons
- ◯ Not sure / didn't specify
- ◯ Other

8. Can you identify reasons why the study may have excluded based on this criteria (tick all that apply)? *

- ☐ Cost of translator services/translation of documents
- ☐ Time taken for translation of documents
- ☐ Availability of interpreters
- ☐ Time pressured nature of the consent process
- ☐ Use of outcome instruments not validated in other languages
- ☐ Advised by sponsor / decision by CTU to exclude
- ☐ Advised by REC
- ☐ Advised by HRA
- ☐ Not sure / can't remember
- ☐ Other

9. Do you believe that exclusion of these participants raises any issues? *

◯ Yes

◯ No

◯ Maybe

10. Please can you provide further details on your answer to the previous question? (e.g. suggest possible issues)

11. Are you aware of initiatives or ideas to improve access to research for those who cannot understand English? (If yes, we would be grateful if you can provide more information in the next question) *

◯ Yes

◯ No

12. Is there anything you would like to add to any of your answers, or provide further information?

Thank you!

Thank you for taking the time to complete our survey, if you have any questions or would like to find out more information, please email rachelle.sherman@nhs.net (mailto:rachelle.sherman@nhs.net).

We will conduct the prize draw a couple of weeks after we close the survey, in April 2021.

13. Would you like to take part in the prize draw to win one of three £20 Amazon vouchers? *

◯ Yes, please

◯ No, thank you

14. If you answered "yes" , please provide your email address below

This content is neither created nor endorsed by Microsoft. The data you submit will be sent to the form owner.

Microsoft Forms
