# Supplementary material for: English language ability as an exclusion criterion in emergency research taking place in the East Midlands, its impact on recruitment and a survey of the views of research professionals
Source: Trials. 2026 Jun 15;27:543. doi: 10.1186/s13063-026-09829-7 (PMC13495170; doi:10.1186/s13063-026-09829-7)
Supplement: Supplementary file 2 [file 13063_2026_9829_MOESM2_ESM.pdf]

Additional File 2

The University Hospitals of Derby & Burton (UHDB) Facilities Management Department provided information on costs associated with the use of translation and interpreter services. Costs linked to units of time (minutes or hours depending on the service) were provided and we used this to estimate the cost of providing interpreters for consent discussions. Similarly, document translation costs were provided "per word" and these were used to estimate an approximate cost to translate commonly seen lengths of patient information leaflets and consent forms.

Outline of costs (excluding VAT) for interpreter and translation services.

**Interpreter/Translation Service Costs**	
Telephone interpretation services	Audio: £0.55 per minute
	Video: £0.75 per minute
Face to face interpretation	£23 per hour
British Sign Language (BSL) interpreter	£100 for a minimum of 2 hours
Document translation (including proofreading)	£0.10 per word, per language.
